# Bis(2-carb­oxy-*N*-{[1-(2-hy­droxy­eth­yl)-3,3-dimethyl­indolin-2-yl­idene]methyl­imino}­anilinium) sulfate monohydrate

**DOI:** 10.1107/S1600536813003188

**Published:** 2013-02-06

**Authors:** Graeme J. Gainsford, Mohamed Ashraf, Andrew J. Kay

**Affiliations:** a Carbohydrate Chemistry Group, Industrial Research Limited, PO Box 31-310, Lower Hutt, New Zealand; b Photonics Group, Industrial Research Limited, PO Box 31-310, Lower Hutt, New Zealand

## Abstract

The asymmetric unit of the title compound, 2C_20_H_22_N_3_O_3_^+^·SO_4_^2−^·H_2_O, contains four cations, two sulfate anions and two lattice water mol­ecules. One of the four cations shows a different conformation of the hy­droxy­ethyl group; the remaining three are all essentially superimposable. Two cations exhibit two-site orientational disorder [ratios = 0.524 (5):0.476 (5) and 0.616 (6):0.384 (6)] of the last two atoms of their hy­droxy­ethyl groups, and one water mol­ecule is disordered over two positions in a 0.634 (13):0.366 (13) ratio. Each imine H atom is intra­molecularly in contact with the adjacent carboxyl O atom, forming an *S*(6) motif, while all the carb­oxy­lic acid H atoms are hydrogen bonded to O atoms of the sulfate anions. Other notable hydrogen-bond inter­actions involve (methyl­ene, phenyl and imine chain) C—H⋯O (sulfate and carbox­yl) and O—H⋯O(water) contacts, making up a comprehensive three-dimensional network involving *D*_2_^2^(*n*), with *n* = 4–6 and 15–16, and *C*_2_^2^(17) classical hydrogen-bond motifs. The crystal investigated was twinned by pseudomerohedry with a twin component ratio of 0.4745 (12):0.5255 (12).

## Related literature

For details of a related synthesis, see: Bhuiyan *et al.* (2011 ▶). For a closely related structure, see: Gainsford *et al.* (2013 ▶). For hydrogen-bonding motifs, see: Bernstein *et al.* (1995 ▶).
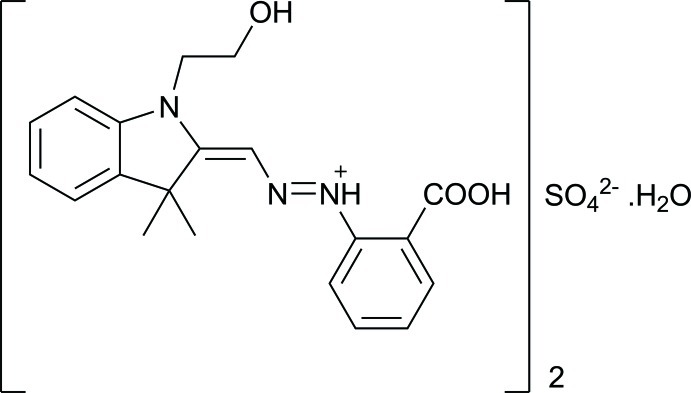


## Experimental

### 

#### Crystal data

2C_20_H_22_N_3_O_3_^+^·SO_4_^2−^·H_2_O
*M* *_r_* = 816.88
Triclinic, 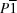

*a* = 12.2530 (9) Å
*b* = 14.6114 (3) Å
*c* = 23.2442 (4) Å
α = 71.681 (1)°
β = 87.688 (2)°
γ = 82.627 (7)°
*V* = 3917.9 (3) Å^3^
*Z* = 4
Cu *K*α radiation
μ = 1.33 mm^−1^
*T* = 153 K
0.68 × 0.40 × 0.24 mm

#### Data collection

Rigaku Spider diffractometer
Absorption correction: multi-scan (*ABSCOR*; Higashi, 1995 ▶) *T*_min_ = 0.66, *T*_max_ = 1.0
24005 measured reflections
10558 independent reflections
9283 reflections with *I* > 2σ(*I*)
*R*_int_ = 0.044
θ_max_ = 58.9°

#### Refinement

*R*[*F* ^2^ > 2σ(*F* ^2^)] = 0.053
*wR*(*F* ^2^) = 0.152
*S* = 1.10
10558 reflections
1069 parameters
2 restraints
H atoms treated by a mixture of independent and constrained refinement
Δρ_max_ = 0.52 e Å^−3^
Δρ_min_ = −0.53 e Å^−3^

### 

Data collection: *CrystalClear* (Rigaku, 2005 ▶); cell refinement: *FSProcess* in *PROCESS-AUTO* (Rigaku, 1998 ▶); data reduction: *FSProcess* in *PROCESS-AUTO*; program(s) used to solve structure: *SHELXS97* (Sheldrick, 2008 ▶); program(s) used to refine structure: *SHELXL97* (Sheldrick, 2008 ▶); molecular graphics: *WinGX* (Farrugia, 2012 ▶) and *Mercury* (Macrae *et al.*, 2008 ▶); software used to prepare material for publication: *SHELXL97* and *PLATON* (Spek, 2009 ▶).

## Supplementary Material

Crystal structure: contains datablock(s) global, I. DOI: 10.1107/S1600536813003188/wm2718sup1.cif

Structure factors: contains datablock(s) I. DOI: 10.1107/S1600536813003188/wm2718Isup2.hkl

Supplementary material file. DOI: 10.1107/S1600536813003188/wm2718Isup3.cml

Additional supplementary materials:  crystallographic information; 3D view; checkCIF report

## Figures and Tables

**Table 1:** Hydrogen-bond geometry (Å, °)

*D*—H⋯*A*	*D*—H	H⋯*A*	*D*⋯*A*	*D*—H⋯*A*
O32*A*—H3A0⋯O101	0.84	1.94	2.644 (8)	140
O2—H2*O*⋯O14	0.84	1.66	2.457 (5)	158
N1—H1*N*⋯O1	0.70 (5)	2.08 (5)	2.652 (5)	139 (6)
O3—H3*O*⋯O13^i^	0.84	1.97	2.792 (5)	165
N101—H11*N*⋯O101	0.97 (5)	1.90 (5)	2.642 (6)	132 (4)
N201—H21*N*⋯O201	0.90 (5)	1.89 (6)	2.626 (6)	138 (5)
O203—H23*O*⋯O701^ii^	0.84	1.95	2.741 (6)	157
N301—H31*N*⋯O301	0.96 (5)	1.95 (5)	2.654 (5)	129 (4)
O102—H102⋯O21^iii^	0.84	1.68	2.509 (5)	169
O202—H202⋯O13^iv^	0.84	1.81	2.565 (5)	149
O302—H302⋯O22^v^	0.84	1.77	2.562 (6)	157
C8—H8⋯O11^vi^	0.95	2.55	3.402 (6)	150
C12*B*—H12*C*⋯O301	0.99	2.41	3.37 (2)	164
C15—H15⋯O12^i^	0.95	2.29	3.143 (6)	149
C19—H19*B*⋯O11^vi^	0.99	2.39	3.357 (7)	166
C20—H20*B*⋯O201^vi^	0.99	2.41	3.343 (6)	157
C220—H22*B*⋯O1^vi^	0.99	2.48	3.456 (6)	168
C39—H39*B*⋯O23	0.99	2.53	3.514 (7)	175
C108—H108⋯O24	0.95	2.26	3.073 (6)	143
C114—H114⋯O102^vii^	0.95	2.54	3.323 (7)	139
C115—H115⋯O23^v^	0.95	2.39	3.202 (7)	143
C205—H205⋯O21^v^	0.95	2.57	3.359 (7)	141
C208—H208⋯O12	0.95	2.26	3.119 (5)	150
C212—H212⋯O80*A*	0.95	2.60	3.486 (10)	156
C215—H215⋯O11^vi^	0.95	2.27	3.137 (7)	152
C306—H306⋯O203^viii^	0.95	2.36	3.154 (7)	140
C308—H308⋯O23	0.95	2.39	3.278 (6)	155
C315—H315⋯O24^iii^	0.95	2.26	3.150 (6)	156

Symmetry codes: (i) 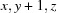; (ii) 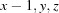; (iii) 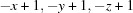; (iv) 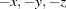; (v) 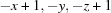; (vi) 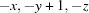; (vii) 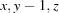; (viii) 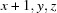.

**Fig. 1.**
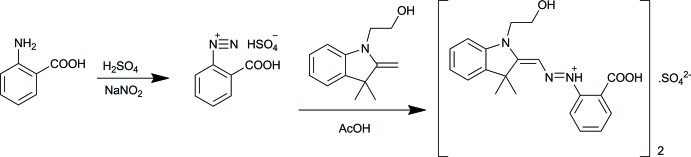
Synthesis method for the title compound.

**Fig. 2.**
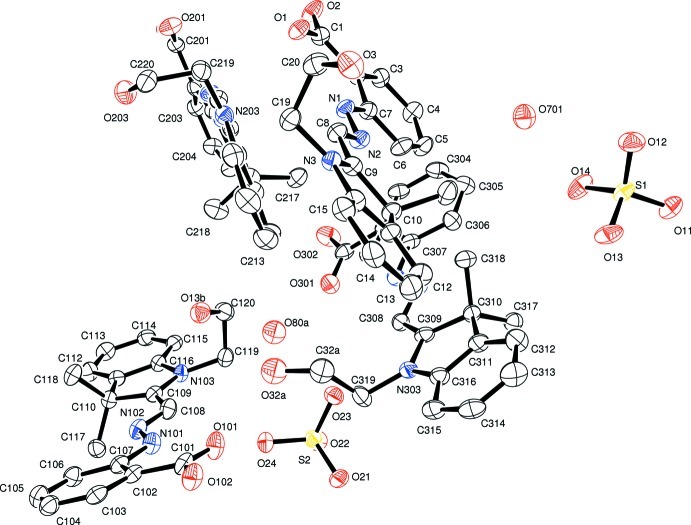
The atoms in the asymmetric unit with 35% probability ellipsoids. Some labels and all hydrogen atoms are omitted for clarity. Only the major conformers of the two site ethyl alcohol atoms are shown (see text).

## supplementary crystallographic information

### Comment

The title compound was prepared as part of our research into novel nonlinear optical (NLO) materials (Bhuiyan *et al.*, 2011). It crystallizes with four independent C_20_H_22_N_3_O_3_^+^ cationic molecules, two sulfate anions and two lattice water molecules in the asymmetric unit (Fig. 2).

Each cation has atom labels related by the addition of 10 (*e.g.* N2, N102, N202, N302), hereafter assigned molecules 1, 2, 3 & 4. Three of the cations (1, 2 & 4) have identical conformations while molecule 3 has a different conformation for the bound hydroxyethyl group. In molecules 2 & 4 there is two-position disorder at the hydroxyethyl group: the partitioning is 0.476 (5):0.524 (5) and 0.616 (6):0.384 (6) for A:B sites in the respective cations. One of the water molecules (O80) is disordered over two sites (0.634 (13):0.366 (13)). The three similar conformation cations (1, 2 & 4) are essentially superimposable, except for the disordered hydroxyethyl atoms. There is bond length agreement (within experimental error) for the four cations, and also with those in a related uncharged structure: bis[(2-{[1-(2-hydroxy-ethyl)-3,3-dimethyl-1,3-dihydro-indol-2-ylidenemethyl]- azo}-benzoic acid)]^.^3H_2_O (Gainsford *et al.*, 2013).

Each imine (N—H) cationic proton is in contact intramolecularly with the adjacent carboxyl oxygen (with a S(6) motif; Bernstein *et al.*, 1995), while all the carboxylic acid protons are hydrogen bonded to O atoms of the sulfate anions. Other notable hydrogen bonds (Table 1) involve (methylene, phenyl & imine chain) C—H···O (sulfate and carboxyl) and O—H···O(water) contacts, making up a comprehensive three-dimensional network involving D^2^_2_(*n*), n = 4–6,15–16, and C^2^_2_(17) classical bond motifs.

### Experimental

To conc. sulfuric acid (4 ml) was added 2-aminobenzoic acid (2.5 mmol) and the reaction mixture cooled to 273–278 K. A solution of sodium nitrite (206 mg, 3 mmol) in 2 ml of water was then slowly added and the reaction stirred at 273–278 K for 30 min. To this was added a solution of 1-hydroxyethyl-3,3-trimethyl-2-methyleneindoline (2 mmol) in 10 ml of glacial acetic acid and the solution stirred for 2 h and allowed to gradually warm to room temperature (Fig. 1). At this point a solid was evident in the reaction mixture. This was collected by filtration, washed with water, dried and recrystallized from ethanol to give the title compound as orange crystals. The filtrate was then neutralized with aqueous sodium carbonate. This resulted in the formation of a second precipitate which after filtration, washing water and recrystallization from ethanol gave fine red needles subsequently identified as bis[(2-{[1-(2-hydroxy-ethyl)-3,3-dimethyl-1,3-dihydro-indol-2-ylidenemethyl]- azo}-benzoic acid)]^.^3H_2_O (Gainsford *et al.*, 2013).

### Refinement

Preliminary scans and initial processing indicated a monoclinic space group with cell parameters *a* = 14.6114 (3), *b* = 44.1324 (9), *c* = 12.2530 (8) Å and *β* = 97.373 (7)° [cell volume = 7835.9 (5) Å^3^]. Careful mapping established that there were 16 cations consistent with a final triclinic (halved volume) cell in space group *P*1. The model was converted to this cell using *PLATON* (Spek, 2009). Further analysis with *PLATON* then revealed that the crystal was twinned by pseudo-merohedry according to the matrix (-1 0 0 0 - 1 0 0 - 1 1); final twin components occupancies were 0.4745 (12):0.5255 (12). One water molecule (O701) was resolved while the other (O80A/O80B) was disordered over two main sites; the H atoms on these O atoms could not be located or calculated and so were not included in the refinement. Fixed occupancies for O80A and O80B (0.634 (13):0.366 (13)) were determined and individual isotropic *U* values were refined. Two site (conformational) disorder was also noted for the terminal C and O atoms of the ethyl alcohol groups on the second cation (C13 & O13) and the fourth cation (C32 & O32), with final occupancies of 0.476 (5):0.524 (5) and 0.616 (6):0.384 (6), respectively. Linked individual isotropic *U* values were refined for these atoms; H atoms on minor conformation atom C32B were fixed in calculated positions in the final cycles of refinement.

A total of 19 weak individual outlier reflections were omitted (using *SHELXL OMIT*) as outliers (Δ(*F*^2^)/e.s.d.(*F*^2^)> 6.2) as shown in the attached. RES file to give the final *R*1 of 0.053. Remaining poor agreement data were all measured as weak or unobserved at high resolution, with *F_o_*^2^ << Fc^2^. There are 241 reflections missing within the final 0.90 Å shell with 26 low angle reflections affected by backstop interactions. An extinction parameter was refined.

All hydroxyl H atoms were constrained to an ideal geometry (0.84 Å) at the difference Fourier map positions (AFIX 147) with *U*_iso_(H) = 1.2*U*_eq_(O). The methyl H atoms were constrained to an ideal geometry (C—H = 0.98 Å) with *U*_iso_(H) = 1.5*U*_eq_(C), but were allowed to rotate freely about the adjacent C—C bond. Imine (N—H) protons were refined using their difference Fourier map assigned positions with *U*_iso_(H) = 1.2*U*_eq_(N). All other H atoms were placed in geometrically idealized positions and constrained o ride on their parent atoms with C—H distances of 1.00 (primary), 0.99 (methylene) or 0.95 (phenyl) Å with *U*_iso_(H) = 1.2*U*_eq_(C) except for H atoms on disordered atoms for which *U*_iso_(H) = 1.5*U*_eq_(C,*O*).

### Crystal data

**Table:**

2C_20_H_22_N_3_O_3_^+^·SO_4_^2^^−^·H_2_O	*Z* = 4
*M**_r_* = 816.88	*F*(000) = 1724
Triclinic, *P*1	*D*_x_ = 1.388 Mg m^−^^3^
Hall symbol: -P 1	Cu *K*α radiation, λ = 1.54178 Å
*a* = 12.2530 (9) Å	Cell parameters from 11451 reflections
*b* = 14.6114 (3) Å	θ = 6.7–71.9°
*c* = 23.2442 (4) Å	µ = 1.33 mm^−^^1^
α = 71.681 (1)°	*T* = 153 K
β = 87.688 (2)°	Needle, orange
γ = 82.627 (7)°	0.68 × 0.40 × 0.24 mm
*V* = 3917.9 (3) Å^3^	

### Data collection

**Table:**

Rigaku Spider diffractometer	10558 independent reflections
Radiation source: Rigaku MM007 rotating anode	9283 reflections with *I* > 2σ(*I*)
Rigaku VariMax-HF Confocal Optical System monochromator	*R*_int_ = 0.044
Detector resolution: 10 pixels mm^-1^	θ_max_ = 58.9°, θ_min_ = 6.7°
ω–scans	*h* = −13→13
Absorption correction: multi-scan (*ABSCOR*; Higashi, 1995)	*k* = −16→12
*T*_min_ = 0.66, *T*_max_ = 1.0	*l* = −25→16
24005 measured reflections	

### Refinement

**Table:**

Refinement on *F*^2^	Secondary atom site location: difference Fourier map
Least-squares matrix: full	Hydrogen site location: difference Fourier map
*R*[*F*^2^ > 2σ(*F*^2^)] = 0.053	H atoms treated by a mixture of independent and constrained refinement
*wR*(*F*^2^) = 0.152	*w* = 1/[σ^2^(*F*_o_^2^) + (0.0892*P*)^2^ + 0.4584*P*] where *P* = (*F*_o_^2^ + 2*F*_c_^2^)/3
*S* = 1.10	(Δ/σ)_max_ < 0.001
10558 reflections	Δρ_max_ = 0.52 e Å^−^^3^
1069 parameters	Δρ_min_ = −0.53 e Å^−^^3^
2 restraints	Extinction correction: *SHELXL97* (Sheldrick, 2008), Fc^*^=kFc[1+0.001xFc^2^λ^3^/sin(2θ)]^-1/4^
Primary atom site location: structure-invariant direct methods	Extinction coefficient: 0.00137 (15)

### Special details

**Table:**

Geometry. All e.s.d.'s (except the e.s.d. in the dihedral angle between two l.s. planes) are estimated using the full covariance matrix. The cell e.s.d.'s are taken into account individually in the estimation of e.s.d.'s in distances, angles and torsion angles; correlations between e.s.d.'s in cell parameters are only used when they are defined by crystal symmetry. An approximate (isotropic) treatment of cell e.s.d.'s is used for estimating e.s.d.'s involving l.s. planes.
Refinement. Refined as a 2-component twin.Reflections were merged by *SHELXL* according to the crystal class for the calculation of statistics and refinement._reflns_Friedel_fraction is defined as the number of unique Friedel pairs measured divided by the number that would be possible theoretically, ignoring centric projections and systematic absences. These statistics refer to single and composite reflections containing twin component 1 only.Refinement of *F*^2^ against ALL reflections. The weighted *R*-factor *wR* and goodness of fit *S* are based on *F*^2^, conventional *R*-factors *R* are based on *F*, with *F* set to zero for negative *F*^2^. The threshold expression of *F*^2^ > σ(*F*^2^) is used only for calculating *R*-factors(gt) *etc*. and is not relevant to the choice of reflections for refinement. *R*-factors based on *F*^2^ are statistically about twice as large as those based on *F*, and *R*- factors based on ALL data will be even larger.

### Fractional atomic coordinates and isotropic or equivalent isotropic displacement parameters (Å^2^)

**Table:**

	*x*	*y*	*z*	*U*_iso_*/*U*_eq_	Occ. (<1)
O1	0.2394 (2)	0.4953 (2)	0.00067 (15)	0.0425 (8)	
O2	0.2862 (3)	0.3439 (2)	−0.00003 (18)	0.0550 (10)	
H2O	0.2244	0.3538	−0.0167	0.082*	
O3	0.2575 (3)	1.0269 (3)	−0.06476 (17)	0.0570 (9)	
H3O	0.2137	1.0733	−0.0601	0.086*	
N1	0.3661 (3)	0.5659 (3)	0.06213 (19)	0.0381 (10)	
H1N	0.321 (4)	0.573 (4)	0.043 (2)	0.046*	
N2	0.3953 (3)	0.6389 (3)	0.07792 (17)	0.0357 (9)	
N3	0.2953 (3)	0.8848 (3)	0.06109 (16)	0.0349 (9)	
C1	0.3040 (4)	0.4204 (4)	0.0130 (2)	0.0380 (11)	
C2	0.4078 (3)	0.4070 (3)	0.0485 (2)	0.0349 (10)	
C3	0.4809 (4)	0.3241 (3)	0.0577 (2)	0.0405 (11)	
H3	0.4641	0.2765	0.0405	0.049*	
C4	0.5765 (4)	0.3060 (4)	0.0902 (2)	0.0480 (13)	
H4	0.6241	0.2470	0.0966	0.058*	
C5	0.6008 (4)	0.3790 (4)	0.1137 (2)	0.0483 (13)	
H5	0.6672	0.3698	0.1356	0.058*	
C6	0.5311 (4)	0.4627 (4)	0.1057 (2)	0.0452 (12)	
H6	0.5481	0.5101	0.1229	0.054*	
C7	0.4349 (3)	0.4786 (3)	0.0721 (2)	0.0355 (11)	
C8	0.3284 (4)	0.7187 (3)	0.0620 (2)	0.0367 (11)	
H8	0.2621	0.7230	0.0410	0.044*	
C9	0.3571 (4)	0.8002 (3)	0.0770 (2)	0.0367 (11)	
C10	0.4570 (4)	0.8038 (3)	0.1123 (2)	0.0353 (10)	
C11	0.4386 (4)	0.9097 (3)	0.1117 (2)	0.0395 (11)	
C12	0.5016 (4)	0.9617 (3)	0.1349 (2)	0.0446 (12)	
H12	0.5693	0.9326	0.1549	0.053*	
C13	0.4631 (4)	1.0566 (4)	0.1279 (3)	0.0521 (14)	
H13	0.5034	1.0926	0.1454	0.063*	
C14	0.3677 (4)	1.1021 (4)	0.0962 (2)	0.0483 (13)	
H14	0.3453	1.1689	0.0912	0.058*	
C15	0.3037 (4)	1.0507 (3)	0.0714 (2)	0.0421 (11)	
H15	0.2374	1.0801	0.0499	0.050*	
C16	0.3435 (4)	0.9549 (3)	0.0803 (2)	0.0364 (11)	
C17	0.5652 (4)	0.7833 (4)	0.0802 (3)	0.0527 (14)	
H17A	0.5623	0.8254	0.0380	0.079*	
H17B	0.5755	0.7151	0.0813	0.079*	
H17C	0.6268	0.7962	0.1009	0.079*	
C18	0.4534 (4)	0.7345 (3)	0.1778 (2)	0.0433 (12)	
H18A	0.4595	0.6673	0.1772	0.065*	
H18B	0.3836	0.7501	0.1968	0.065*	
H18C	0.5147	0.7421	0.2010	0.065*	
C19	0.1909 (4)	0.9098 (4)	0.0259 (2)	0.0441 (12)	
H19A	0.1454	0.9636	0.0362	0.053*	
H19B	0.1493	0.8529	0.0380	0.053*	
C20	0.2093 (4)	0.9395 (4)	−0.0415 (2)	0.0503 (13)	
H20A	0.2575	0.8868	−0.0514	0.060*	
H20B	0.1378	0.9473	−0.0619	0.060*	
O101	0.2709 (3)	0.5022 (2)	0.49747 (19)	0.0560 (10)	
O102	0.2103 (3)	0.6547 (2)	0.49437 (19)	0.0532 (9)	
H102	0.2728	0.6648	0.4794	0.080*	
N101	0.1521 (3)	0.3627 (3)	0.55596 (19)	0.0436 (10)	
H11N	0.222 (4)	0.380 (3)	0.538 (2)	0.052*	
N102	0.1256 (3)	0.2723 (3)	0.56968 (19)	0.0423 (10)	
N103	0.2293 (3)	0.0462 (2)	0.54970 (17)	0.0341 (9)	
C101	0.2017 (4)	0.5614 (4)	0.5104 (2)	0.0486 (13)	
C102	0.1027 (4)	0.5326 (3)	0.5464 (2)	0.0394 (11)	
C103	0.0256 (4)	0.6039 (4)	0.5589 (2)	0.0467 (13)	
H103	0.0384	0.6700	0.5436	0.056*	
C104	−0.0675 (4)	0.5810 (4)	0.5924 (2)	0.0453 (12)	
H104	−0.1196	0.6308	0.5989	0.054*	
C105	−0.0849 (4)	0.4839 (4)	0.6169 (2)	0.0516 (13)	
H105	−0.1476	0.4676	0.6416	0.062*	
C106	−0.0125 (4)	0.4118 (4)	0.6056 (2)	0.0463 (13)	
H106	−0.0260	0.3461	0.6225	0.056*	
C107	0.0807 (4)	0.4335 (4)	0.5697 (2)	0.0427 (12)	
C108	0.1946 (4)	0.2094 (3)	0.5532 (2)	0.0446 (12)	
H108	0.2614	0.2270	0.5331	0.054*	
C109	0.1666 (4)	0.1144 (3)	0.5662 (2)	0.0353 (10)	
C110	0.0659 (3)	0.0743 (3)	0.6005 (2)	0.0343 (10)	
C111	0.0845 (4)	−0.0269 (3)	0.5995 (2)	0.0371 (11)	
C112	0.0219 (4)	−0.1025 (4)	0.6212 (2)	0.0464 (13)	
H112	−0.0458	−0.0938	0.6414	0.056*	
C113	0.0593 (4)	−0.1928 (3)	0.6132 (2)	0.0475 (13)	
H113	0.0169	−0.2454	0.6286	0.057*	
C114	0.1566 (4)	−0.2060 (4)	0.5833 (2)	0.0459 (12)	
H114	0.1804	−0.2675	0.5784	0.055*	
C115	0.2206 (4)	−0.1303 (4)	0.5603 (2)	0.0463 (12)	
H115	0.2877	−0.1389	0.5396	0.056*	
C116	0.1818 (3)	−0.0412 (3)	0.5689 (2)	0.0354 (10)	
C117	0.0632 (4)	0.0801 (4)	0.6656 (2)	0.0486 (13)	
H11A	−0.0007	0.0513	0.6870	0.073*	
H11B	0.0579	0.1482	0.6642	0.073*	
H11C	0.1307	0.0444	0.6869	0.073*	
C118	−0.0400 (4)	0.1281 (3)	0.5662 (3)	0.0485 (13)	
H11D	−0.0331	0.1302	0.5237	0.073*	
H11E	−0.0517	0.1945	0.5685	0.073*	
H11F	−0.1027	0.0940	0.5846	0.073*	
C119	0.3262 (4)	0.0599 (4)	0.5101 (2)	0.0444 (12)	
H11G	0.3783	−0.0006	0.5218	0.053*	
H11H	0.3636	0.1124	0.5160	0.053*	
O201	0.0154 (3)	−0.0296 (3)	0.12762 (16)	0.0549 (9)	
O202	0.0235 (3)	−0.1928 (2)	0.15901 (16)	0.0527 (9)	
H202	0.0056	−0.1860	0.1232	0.079*	
O203	−0.0898 (3)	0.3446 (3)	0.15518 (17)	0.0623 (10)	
H23O	−0.1143	0.3162	0.1330	0.093*	
N201	0.1403 (3)	0.0481 (3)	0.18402 (19)	0.0429 (10)	
H21N	0.100 (4)	0.051 (4)	0.152 (2)	0.052*	
N202	0.1727 (3)	0.1232 (3)	0.19590 (19)	0.0431 (10)	
N203	0.1385 (3)	0.3820 (3)	0.14242 (18)	0.0403 (9)	
C201	0.0471 (4)	−0.1083 (4)	0.1628 (2)	0.0416 (12)	
C202	0.1215 (4)	−0.1230 (3)	0.2154 (2)	0.0370 (11)	
C203	0.1466 (4)	−0.2138 (4)	0.2579 (2)	0.0464 (12)	
H203	0.1145	−0.2671	0.2535	0.056*	
C204	0.2158 (4)	−0.2302 (4)	0.3060 (2)	0.0511 (13)	
H204	0.2305	−0.2932	0.3345	0.061*	
C205	0.2640 (4)	−0.1524 (4)	0.3124 (3)	0.0545 (14)	
H205	0.3133	−0.1626	0.3449	0.065*	
C206	0.2403 (4)	−0.0616 (4)	0.2718 (2)	0.0503 (13)	
H206	0.2728	−0.0089	0.2768	0.060*	
C207	0.1689 (4)	−0.0452 (3)	0.2232 (2)	0.0395 (11)	
C208	0.1423 (4)	0.2094 (4)	0.1587 (2)	0.0456 (12)	
H208	0.1014	0.2179	0.1232	0.055*	
C209	0.1724 (4)	0.2905 (4)	0.1732 (2)	0.0396 (11)	
C210	0.2454 (4)	0.2845 (3)	0.2258 (2)	0.0380 (11)	
C211	0.2434 (4)	0.3926 (4)	0.2180 (2)	0.0448 (12)	
C212	0.2887 (5)	0.4385 (4)	0.2527 (2)	0.0525 (14)	
H212	0.3337	0.4026	0.2867	0.063*	
C213	0.2670 (5)	0.5395 (4)	0.2368 (3)	0.0587 (15)	
H213	0.2991	0.5726	0.2598	0.070*	
C214	0.2002 (5)	0.5921 (4)	0.1886 (3)	0.0579 (15)	
H214	0.1855	0.6606	0.1794	0.069*	
C215	0.1532 (4)	0.5456 (4)	0.1527 (3)	0.0534 (13)	
H215	0.1082	0.5810	0.1186	0.064*	
C216	0.1763 (4)	0.4451 (3)	0.1697 (2)	0.0387 (11)	
C217	0.3617 (4)	0.2374 (4)	0.2206 (3)	0.0498 (13)	
H21A	0.4055	0.2367	0.2551	0.075*	
H21B	0.3951	0.2745	0.1828	0.075*	
H21C	0.3596	0.1707	0.2204	0.075*	
C218	0.1926 (5)	0.2353 (4)	0.2870 (2)	0.0563 (15)	
H21D	0.1161	0.2646	0.2875	0.084*	
H21E	0.2337	0.2441	0.3198	0.084*	
H21F	0.1942	0.1658	0.2927	0.084*	
C219	0.0597 (4)	0.4180 (4)	0.0927 (2)	0.0484 (13)	
H21G	0.0618	0.3708	0.0700	0.058*	
H21H	0.0786	0.4804	0.0645	0.058*	
C220	−0.0559 (4)	0.4327 (4)	0.1189 (2)	0.0582 (15)	
H22A	−0.0560	0.4772	0.1434	0.070*	
H22B	−0.1088	0.4635	0.0853	0.070*	
O301	0.5326 (3)	0.0855 (2)	0.37373 (16)	0.0484 (9)	
O302	0.5300 (4)	−0.0437 (2)	0.34318 (18)	0.0624 (10)	
H302	0.4828	−0.0580	0.3708	0.094*	
N301	0.6508 (3)	0.2239 (3)	0.31096 (19)	0.0410 (9)	
H31N	0.596 (4)	0.207 (3)	0.342 (2)	0.049*	
N302	0.6845 (3)	0.3113 (3)	0.29572 (18)	0.0408 (9)	
N303	0.6494 (3)	0.5197 (3)	0.34567 (17)	0.0380 (9)	
C301	0.5578 (4)	0.0421 (3)	0.3380 (2)	0.0409 (11)	
C302	0.6339 (4)	0.0764 (3)	0.2857 (2)	0.0378 (11)	
C303	0.6614 (4)	0.0227 (3)	0.2459 (2)	0.0446 (12)	
H303	0.6264	−0.0338	0.2511	0.054*	
C304	0.7359 (5)	0.0471 (4)	0.1999 (2)	0.0533 (14)	
H304	0.7516	0.0095	0.1732	0.064*	
C305	0.7887 (4)	0.1296 (4)	0.1935 (2)	0.0523 (13)	
H305	0.8435	0.1463	0.1632	0.063*	
C306	0.7623 (4)	0.1868 (4)	0.2305 (2)	0.0467 (12)	
H306	0.7985	0.2425	0.2255	0.056*	
C307	0.6819 (4)	0.1626 (3)	0.2753 (2)	0.0405 (11)	
C308	0.6496 (4)	0.3640 (3)	0.3307 (2)	0.0382 (11)	
H308	0.6034	0.3406	0.3645	0.046*	
C309	0.6842 (4)	0.4577 (3)	0.3155 (2)	0.0393 (11)	
C310	0.7633 (4)	0.5041 (3)	0.2650 (2)	0.0370 (11)	
C311	0.7620 (4)	0.6022 (3)	0.2728 (2)	0.0382 (11)	
C312	0.8157 (4)	0.6804 (3)	0.2401 (3)	0.0520 (14)	
H312	0.8630	0.6767	0.2073	0.062*	
C313	0.7977 (5)	0.7655 (4)	0.2571 (3)	0.0589 (15)	
H313	0.8337	0.8201	0.2356	0.071*	
C314	0.7280 (4)	0.7707 (4)	0.3049 (3)	0.0518 (13)	
H314	0.7176	0.8288	0.3155	0.062*	
C315	0.6736 (4)	0.6939 (3)	0.3371 (2)	0.0463 (13)	
H315	0.6248	0.6982	0.3692	0.056*	
C316	0.6931 (4)	0.6100 (3)	0.3205 (2)	0.0408 (11)	
C317	0.8780 (4)	0.4465 (4)	0.2744 (2)	0.0452 (12)	
H31A	0.9022	0.4348	0.3161	0.068*	
H31B	0.9297	0.4835	0.2460	0.068*	
H31C	0.8755	0.3842	0.2672	0.068*	
C318	0.7170 (4)	0.5133 (4)	0.2029 (2)	0.0448 (12)	
H31D	0.7202	0.4486	0.1983	0.067*	
H31E	0.7609	0.5535	0.1709	0.067*	
H31F	0.6405	0.5436	0.1998	0.067*	
S1	0.06408 (9)	0.24937 (9)	−0.03304 (6)	0.0409 (3)	
O11	−0.0552 (3)	0.2821 (2)	−0.04153 (16)	0.0511 (9)	
O12	0.0915 (3)	0.2003 (3)	0.03033 (15)	0.0565 (9)	
O13	0.0984 (3)	0.1842 (2)	−0.06986 (16)	0.0544 (9)	
O14	0.1239 (3)	0.3376 (2)	−0.05672 (17)	0.0568 (10)	
S2	0.54556 (9)	0.23700 (8)	0.53390 (5)	0.0383 (3)	
O21	0.6139 (3)	0.2948 (2)	0.55787 (15)	0.0459 (8)	
O22	0.5804 (3)	0.1314 (2)	0.56443 (16)	0.0493 (8)	
O23	0.5641 (3)	0.2580 (3)	0.46818 (15)	0.0506 (9)	
O24	0.4308 (2)	0.2617 (3)	0.54699 (16)	0.0550 (9)	
O701	0.8376 (3)	0.2999 (3)	0.05839 (18)	0.0680 (11)	
O13A	0.2507 (6)	0.0149 (5)	0.4261 (4)	0.0556 (13)*	0.476 (5)
H13A	0.1859	0.0365	0.4143	0.083*	0.476 (5)
C12A	0.297 (2)	0.0848 (19)	0.4455 (14)	0.0506 (18)*	0.476 (5)
H12A	0.2444	0.1449	0.4351	0.076*	0.476 (5)
H12B	0.3645	0.1005	0.4214	0.076*	0.476 (5)
C39	0.5651 (4)	0.5077 (4)	0.3938 (2)	0.0515 (13)	
H39A	0.5796	0.5433	0.4220	0.077*	
H39B	0.5648	0.4382	0.4171	0.077*	
C32A	0.4486 (11)	0.5519 (10)	0.3593 (6)	0.0733 (14)*	0.616 (6)
H32A	0.4388	0.6231	0.3513	0.110*	0.616 (6)
H32B	0.4523	0.5399	0.3196	0.110*	0.616 (6)
O32A	0.3578 (6)	0.5154 (5)	0.3896 (3)	0.0733 (14)*	0.616 (6)
H3AO	0.3457	0.5353	0.4198	0.110*	0.616 (6)
O80A	0.3757 (7)	0.3100 (7)	0.4002 (4)	0.0739 (17)*	0.634 (13)
O80B	0.3461 (12)	0.3430 (11)	0.4156 (6)	0.0739 (17)*	0.366 (13)
O13B	0.2195 (5)	0.1859 (5)	0.4300 (3)	0.0556 (13)*	0.524 (5)
H13B	0.2591	0.2311	0.4225	0.0739 (17)*	0.524 (5)
C12B	0.2868 (19)	0.0965 (17)	0.4437 (12)	0.0506 (18)*	0.524 (5)
H12C	0.3516	0.1035	0.4165	0.076*	0.524 (5)
H12D	0.2453	0.0476	0.4360	0.076*	0.524 (5)
O32B	0.4336 (10)	0.6167 (9)	0.3458 (5)	0.0733 (14)*	0.384 (6)
H3BO	0.4092	0.6486	0.3690	0.110*	0.384 (6)
C32B	0.4624 (18)	0.5261 (15)	0.3778 (9)	0.0733 (14)*	0.384 (6)
H32C	0.4158	0.5106	0.4149	0.110*	0.384 (6)
H32D	0.4468	0.4835	0.3543	0.110*	0.384 (6)

### Atomic displacement parameters (Å^2^)

**Table:**

	*U*^11^	*U*^22^	*U*^33^	*U*^12^	*U*^13^	*U*^23^
O1	0.0406 (17)	0.0372 (19)	0.054 (2)	−0.0068 (16)	−0.0109 (15)	−0.0182 (16)
O2	0.0453 (19)	0.045 (2)	0.088 (3)	−0.0001 (16)	−0.0196 (19)	−0.040 (2)
O3	0.065 (2)	0.046 (2)	0.058 (2)	−0.0045 (18)	0.0084 (19)	−0.0147 (19)
N1	0.036 (2)	0.041 (2)	0.043 (3)	−0.0037 (19)	−0.0079 (18)	−0.020 (2)
N2	0.044 (2)	0.027 (2)	0.045 (2)	−0.0061 (18)	−0.0011 (18)	−0.0223 (18)
N3	0.0340 (19)	0.038 (2)	0.036 (2)	−0.0026 (17)	−0.0020 (16)	−0.0168 (18)
C1	0.035 (2)	0.038 (3)	0.048 (3)	−0.009 (2)	0.001 (2)	−0.021 (2)
C2	0.036 (2)	0.032 (2)	0.038 (3)	−0.006 (2)	0.001 (2)	−0.012 (2)
C3	0.043 (3)	0.034 (3)	0.050 (3)	−0.012 (2)	0.002 (2)	−0.018 (2)
C4	0.044 (3)	0.044 (3)	0.056 (3)	0.000 (2)	−0.006 (2)	−0.018 (3)
C5	0.037 (3)	0.058 (3)	0.058 (3)	−0.009 (2)	−0.008 (2)	−0.028 (3)
C6	0.048 (3)	0.040 (3)	0.055 (3)	0.002 (2)	−0.014 (2)	−0.025 (3)
C7	0.037 (2)	0.029 (3)	0.042 (3)	−0.005 (2)	0.000 (2)	−0.014 (2)
C8	0.040 (2)	0.032 (3)	0.044 (3)	−0.003 (2)	−0.005 (2)	−0.020 (2)
C9	0.040 (2)	0.032 (3)	0.037 (3)	−0.004 (2)	0.003 (2)	−0.010 (2)
C10	0.042 (2)	0.027 (2)	0.041 (3)	−0.009 (2)	−0.001 (2)	−0.015 (2)
C11	0.044 (3)	0.040 (3)	0.042 (3)	−0.014 (2)	0.002 (2)	−0.021 (2)
C12	0.046 (3)	0.039 (3)	0.060 (3)	−0.004 (2)	0.000 (2)	−0.032 (3)
C13	0.051 (3)	0.052 (3)	0.068 (4)	−0.019 (3)	0.011 (3)	−0.035 (3)
C14	0.054 (3)	0.036 (3)	0.060 (4)	−0.008 (2)	0.015 (3)	−0.022 (3)
C15	0.045 (3)	0.034 (3)	0.045 (3)	−0.002 (2)	0.002 (2)	−0.011 (2)
C16	0.043 (3)	0.023 (2)	0.048 (3)	−0.011 (2)	0.007 (2)	−0.015 (2)
C17	0.037 (3)	0.061 (3)	0.070 (4)	−0.009 (2)	0.003 (3)	−0.033 (3)
C18	0.053 (3)	0.034 (3)	0.046 (3)	−0.007 (2)	−0.004 (2)	−0.016 (2)
C19	0.035 (2)	0.046 (3)	0.052 (3)	−0.003 (2)	−0.007 (2)	−0.015 (2)
C20	0.050 (3)	0.045 (3)	0.052 (3)	0.006 (2)	−0.014 (2)	−0.012 (3)
O101	0.0378 (18)	0.039 (2)	0.084 (3)	−0.0081 (16)	0.0055 (19)	−0.0087 (19)
O102	0.049 (2)	0.0308 (19)	0.083 (3)	−0.0166 (15)	0.0041 (19)	−0.0187 (18)
N101	0.040 (2)	0.034 (2)	0.054 (3)	−0.0032 (19)	0.001 (2)	−0.012 (2)
N102	0.039 (2)	0.032 (2)	0.052 (3)	−0.0088 (18)	−0.0055 (19)	−0.0050 (19)
N103	0.0327 (19)	0.024 (2)	0.050 (2)	−0.0058 (16)	0.0011 (17)	−0.0157 (18)
C101	0.031 (3)	0.058 (4)	0.051 (3)	−0.007 (3)	−0.010 (2)	−0.008 (3)
C102	0.037 (2)	0.041 (3)	0.041 (3)	−0.013 (2)	−0.008 (2)	−0.011 (2)
C103	0.042 (3)	0.051 (3)	0.048 (3)	−0.005 (2)	−0.015 (2)	−0.015 (3)
C104	0.042 (3)	0.041 (3)	0.058 (3)	−0.011 (2)	−0.004 (2)	−0.019 (3)
C105	0.044 (3)	0.055 (4)	0.056 (3)	−0.010 (3)	0.002 (2)	−0.016 (3)
C106	0.050 (3)	0.039 (3)	0.058 (3)	−0.021 (2)	0.001 (2)	−0.019 (3)
C107	0.038 (3)	0.045 (3)	0.042 (3)	−0.009 (2)	−0.008 (2)	−0.007 (2)
C108	0.035 (2)	0.044 (3)	0.054 (3)	−0.005 (2)	−0.001 (2)	−0.013 (2)
C109	0.041 (2)	0.026 (2)	0.039 (3)	−0.011 (2)	−0.007 (2)	−0.007 (2)
C110	0.037 (2)	0.019 (2)	0.050 (3)	−0.0015 (19)	−0.002 (2)	−0.015 (2)
C111	0.040 (2)	0.030 (3)	0.038 (3)	−0.002 (2)	−0.004 (2)	−0.006 (2)
C112	0.042 (3)	0.047 (3)	0.052 (3)	−0.017 (2)	0.008 (2)	−0.014 (3)
C113	0.055 (3)	0.031 (3)	0.057 (3)	−0.006 (2)	−0.004 (3)	−0.014 (2)
C114	0.049 (3)	0.037 (3)	0.053 (3)	−0.010 (2)	−0.009 (3)	−0.014 (2)
C115	0.038 (3)	0.061 (3)	0.041 (3)	0.010 (2)	−0.007 (2)	−0.023 (3)
C116	0.035 (2)	0.029 (3)	0.042 (3)	0.0041 (19)	−0.003 (2)	−0.014 (2)
C117	0.045 (3)	0.055 (3)	0.051 (3)	−0.016 (2)	0.006 (2)	−0.022 (3)
C118	0.039 (3)	0.040 (3)	0.065 (4)	−0.003 (2)	−0.007 (2)	−0.014 (3)
C119	0.037 (3)	0.042 (3)	0.055 (3)	−0.005 (2)	0.002 (2)	−0.017 (3)
O201	0.064 (2)	0.048 (2)	0.050 (2)	−0.0064 (18)	−0.0194 (18)	−0.0101 (19)
O202	0.068 (2)	0.051 (2)	0.046 (2)	−0.0135 (18)	−0.0121 (18)	−0.0212 (18)
O203	0.060 (2)	0.066 (3)	0.059 (2)	−0.023 (2)	0.0091 (19)	−0.012 (2)
N201	0.049 (2)	0.039 (2)	0.047 (3)	−0.0161 (19)	−0.006 (2)	−0.017 (2)
N202	0.043 (2)	0.044 (3)	0.048 (3)	−0.0132 (19)	−0.0006 (19)	−0.019 (2)
N203	0.041 (2)	0.035 (2)	0.045 (2)	−0.0029 (18)	0.0012 (19)	−0.0134 (19)
C201	0.046 (3)	0.050 (3)	0.039 (3)	−0.016 (2)	0.004 (2)	−0.025 (3)
C202	0.045 (3)	0.034 (3)	0.038 (3)	−0.014 (2)	0.011 (2)	−0.018 (2)
C203	0.050 (3)	0.048 (3)	0.046 (3)	−0.010 (2)	0.010 (2)	−0.021 (3)
C204	0.058 (3)	0.052 (3)	0.046 (3)	−0.013 (3)	−0.002 (3)	−0.017 (3)
C205	0.056 (3)	0.060 (4)	0.048 (3)	−0.008 (3)	−0.008 (3)	−0.015 (3)
C206	0.053 (3)	0.050 (3)	0.057 (3)	−0.015 (3)	−0.005 (3)	−0.026 (3)
C207	0.042 (3)	0.039 (3)	0.040 (3)	−0.010 (2)	0.005 (2)	−0.016 (2)
C208	0.040 (3)	0.056 (3)	0.043 (3)	−0.006 (2)	−0.001 (2)	−0.019 (3)
C209	0.035 (2)	0.047 (3)	0.043 (3)	−0.007 (2)	0.006 (2)	−0.023 (2)
C210	0.054 (3)	0.022 (2)	0.042 (3)	−0.008 (2)	0.003 (2)	−0.015 (2)
C211	0.048 (3)	0.045 (3)	0.039 (3)	−0.010 (2)	0.008 (2)	−0.008 (2)
C212	0.070 (3)	0.039 (3)	0.052 (3)	−0.002 (3)	−0.009 (3)	−0.019 (3)
C213	0.075 (4)	0.048 (3)	0.063 (4)	−0.014 (3)	0.012 (3)	−0.030 (3)
C214	0.075 (4)	0.033 (3)	0.058 (4)	0.002 (3)	0.013 (3)	−0.009 (3)
C215	0.050 (3)	0.052 (3)	0.052 (3)	−0.005 (3)	0.006 (3)	−0.008 (3)
C216	0.049 (3)	0.020 (2)	0.048 (3)	0.000 (2)	0.008 (2)	−0.014 (2)
C217	0.047 (3)	0.047 (3)	0.060 (3)	−0.003 (2)	−0.014 (3)	−0.023 (3)
C218	0.086 (4)	0.037 (3)	0.046 (3)	−0.014 (3)	0.008 (3)	−0.011 (3)
C219	0.050 (3)	0.046 (3)	0.045 (3)	−0.008 (2)	0.003 (2)	−0.008 (2)
C220	0.046 (3)	0.068 (4)	0.047 (3)	−0.009 (3)	−0.006 (3)	0.003 (3)
O301	0.058 (2)	0.0355 (19)	0.058 (2)	−0.0206 (16)	0.0082 (17)	−0.0188 (18)
O302	0.100 (3)	0.037 (2)	0.059 (3)	−0.034 (2)	0.013 (2)	−0.0200 (18)
N301	0.045 (2)	0.040 (2)	0.045 (2)	−0.0125 (18)	0.0037 (19)	−0.020 (2)
N302	0.045 (2)	0.037 (2)	0.042 (2)	−0.0101 (18)	−0.0037 (18)	−0.012 (2)
N303	0.040 (2)	0.044 (2)	0.036 (2)	−0.0138 (18)	0.0023 (17)	−0.0167 (19)
C301	0.053 (3)	0.026 (2)	0.044 (3)	−0.014 (2)	−0.010 (2)	−0.007 (2)
C302	0.044 (3)	0.028 (3)	0.039 (3)	−0.005 (2)	−0.009 (2)	−0.008 (2)
C303	0.065 (3)	0.028 (3)	0.047 (3)	−0.010 (2)	−0.012 (3)	−0.018 (2)
C304	0.075 (4)	0.048 (3)	0.037 (3)	−0.012 (3)	−0.004 (3)	−0.011 (2)
C305	0.064 (3)	0.052 (3)	0.043 (3)	−0.015 (3)	0.005 (3)	−0.016 (3)
C306	0.051 (3)	0.046 (3)	0.043 (3)	−0.011 (2)	0.005 (2)	−0.012 (3)
C307	0.048 (3)	0.041 (3)	0.034 (3)	−0.011 (2)	−0.006 (2)	−0.011 (2)
C308	0.041 (2)	0.039 (3)	0.035 (3)	−0.011 (2)	0.004 (2)	−0.012 (2)
C309	0.043 (3)	0.042 (3)	0.039 (3)	−0.008 (2)	−0.006 (2)	−0.019 (2)
C310	0.037 (2)	0.043 (3)	0.037 (3)	−0.011 (2)	0.004 (2)	−0.018 (2)
C311	0.046 (3)	0.026 (2)	0.048 (3)	−0.005 (2)	−0.001 (2)	−0.019 (2)
C312	0.066 (3)	0.034 (3)	0.055 (3)	−0.012 (2)	0.010 (3)	−0.011 (3)
C313	0.078 (4)	0.035 (3)	0.067 (4)	−0.016 (3)	−0.003 (3)	−0.018 (3)
C314	0.070 (3)	0.037 (3)	0.057 (3)	−0.007 (3)	−0.009 (3)	−0.027 (3)
C315	0.056 (3)	0.041 (3)	0.050 (3)	0.001 (2)	−0.005 (2)	−0.028 (3)
C316	0.045 (3)	0.041 (3)	0.040 (3)	−0.009 (2)	0.001 (2)	−0.017 (2)
C317	0.041 (3)	0.056 (3)	0.040 (3)	−0.010 (2)	−0.001 (2)	−0.016 (2)
C318	0.049 (3)	0.051 (3)	0.038 (3)	−0.016 (2)	−0.006 (2)	−0.014 (2)
S1	0.0396 (6)	0.0418 (7)	0.0452 (8)	−0.0059 (5)	−0.0093 (5)	−0.0176 (6)
O11	0.0412 (18)	0.048 (2)	0.065 (2)	0.0019 (16)	−0.0161 (17)	−0.0206 (19)
O12	0.058 (2)	0.066 (2)	0.041 (2)	0.0004 (18)	−0.0133 (17)	−0.0116 (19)
O13	0.063 (2)	0.051 (2)	0.063 (2)	0.0035 (17)	−0.0159 (18)	−0.0389 (19)
O14	0.059 (2)	0.037 (2)	0.078 (3)	−0.0220 (17)	−0.0202 (19)	−0.0146 (19)
S2	0.0358 (6)	0.0413 (7)	0.0395 (7)	−0.0087 (5)	0.0016 (5)	−0.0138 (6)
O21	0.0521 (19)	0.0420 (19)	0.055 (2)	−0.0208 (15)	0.0017 (16)	−0.0253 (17)
O22	0.064 (2)	0.0286 (18)	0.052 (2)	−0.0103 (16)	0.0005 (17)	−0.0065 (16)
O23	0.0484 (19)	0.062 (2)	0.0366 (19)	0.0041 (17)	0.0005 (16)	−0.0132 (17)
O24	0.0329 (17)	0.076 (3)	0.060 (2)	−0.0076 (17)	0.0079 (16)	−0.027 (2)
O701	0.060 (2)	0.076 (3)	0.071 (3)	−0.010 (2)	0.002 (2)	−0.027 (2)
C39	0.049 (3)	0.062 (4)	0.045 (3)	−0.012 (3)	0.015 (2)	−0.020 (3)

### Geometric parameters (Å, º)

**Table:**

O1—C1	1.227 (5)	C201—C202	1.500 (7)
O2—C1	1.292 (5)	C202—C203	1.389 (7)
O2—H2O	0.8400	C202—C207	1.403 (6)
O3—C20	1.417 (6)	C203—C204	1.372 (7)
O3—H3O	0.8400	C203—H203	0.9500
N1—N2	1.325 (5)	C204—C205	1.395 (7)
N1—C7	1.395 (6)	C204—H204	0.9500
N1—H1N	0.70 (5)	C205—C206	1.368 (7)
N2—C8	1.295 (5)	C205—H205	0.9500
N3—C9	1.317 (5)	C206—C207	1.399 (7)
N3—C16	1.435 (5)	C206—H206	0.9500
N3—C19	1.488 (5)	C208—C209	1.422 (6)
C1—C2	1.501 (6)	C208—H208	0.9500
C2—C3	1.374 (6)	C209—C210	1.519 (6)
C2—C7	1.404 (6)	C210—C217	1.518 (7)
C3—C4	1.372 (6)	C210—C211	1.530 (6)
C3—H3	0.9500	C210—C218	1.537 (7)
C4—C5	1.409 (7)	C211—C212	1.372 (7)
C4—H4	0.9500	C211—C216	1.376 (7)
C5—C6	1.364 (7)	C212—C213	1.396 (7)
C5—H5	0.9500	C212—H212	0.9500
C6—C7	1.393 (6)	C213—C214	1.373 (8)
C6—H6	0.9500	C213—H213	0.9500
C8—C9	1.427 (6)	C214—C215	1.410 (8)
C8—H8	0.9500	C214—H214	0.9500
C9—C10	1.516 (6)	C215—C216	1.389 (7)
C10—C11	1.530 (6)	C215—H215	0.9500
C10—C17	1.537 (7)	C217—H21A	0.9800
C10—C18	1.545 (6)	C217—H21B	0.9800
C11—C16	1.376 (6)	C217—H21C	0.9800
C11—C12	1.381 (6)	C218—H21D	0.9800
C12—C13	1.366 (7)	C218—H21E	0.9800
C12—H12	0.9500	C218—H21F	0.9800
C13—C14	1.383 (7)	C219—C220	1.535 (7)
C13—H13	0.9500	C219—H21G	0.9900
C14—C15	1.405 (7)	C219—H21H	0.9900
C14—H14	0.9500	C220—H22A	0.9900
C15—C16	1.375 (6)	C220—H22B	0.9900
C15—H15	0.9500	O301—C301	1.204 (5)
C17—H17A	0.9800	O302—C301	1.309 (5)
C17—H17B	0.9800	O302—H302	0.8400
C17—H17C	0.9800	N301—N302	1.330 (5)
C18—H18A	0.9800	N301—C307	1.410 (6)
C18—H18B	0.9800	N301—H31N	0.96 (5)
C18—H18C	0.9800	N302—C308	1.310 (6)
C19—C20	1.504 (7)	N303—C309	1.332 (6)
C19—H19A	0.9900	N303—C316	1.428 (6)
C19—H19B	0.9900	N303—C39	1.476 (6)
C20—H20A	0.9900	C301—C302	1.497 (7)
C20—H20B	0.9900	C302—C303	1.396 (6)
O101—C101	1.230 (6)	C302—C307	1.405 (6)
O102—C101	1.312 (6)	C303—C304	1.368 (7)
O102—H102	0.8400	C303—H303	0.9500
N101—N102	1.336 (5)	C304—C305	1.405 (7)
N101—C107	1.374 (6)	C304—H304	0.9500
N101—H11N	0.97 (5)	C305—C306	1.378 (7)
N102—C108	1.305 (6)	C305—H305	0.9500
N103—C109	1.322 (5)	C306—C307	1.399 (7)
N103—C116	1.407 (5)	C306—H306	0.9500
N103—C119	1.467 (6)	C308—C309	1.418 (6)
C101—C102	1.475 (7)	C308—H308	0.9500
C102—C103	1.406 (7)	C309—C310	1.534 (6)
C102—C107	1.435 (6)	C310—C311	1.497 (6)
C103—C104	1.373 (7)	C310—C317	1.528 (6)
C103—H103	0.9500	C310—C318	1.533 (6)
C104—C105	1.393 (7)	C311—C312	1.387 (7)
C104—H104	0.9500	C311—C316	1.392 (7)
C105—C106	1.371 (7)	C312—C313	1.408 (7)
C105—H105	0.9500	C312—H312	0.9500
C106—C107	1.398 (7)	C313—C314	1.388 (8)
C106—H106	0.9500	C313—H313	0.9500
C108—C109	1.410 (6)	C314—C315	1.374 (7)
C108—H108	0.9500	C314—H314	0.9500
C109—C110	1.521 (6)	C315—C316	1.387 (6)
C110—C111	1.474 (6)	C315—H315	0.9500
C110—C118	1.539 (6)	C317—H31A	0.9800
C110—C117	1.539 (6)	C317—H31B	0.9800
C111—C112	1.379 (6)	C317—H31C	0.9800
C111—C116	1.391 (6)	C318—H31D	0.9800
C112—C113	1.405 (7)	C318—H31E	0.9800
C112—H112	0.9500	C318—H31F	0.9800
C113—C114	1.379 (7)	S1—O12	1.453 (4)
C113—H113	0.9500	S1—O11	1.479 (3)
C114—C115	1.392 (7)	S1—O13	1.482 (3)
C114—H114	0.9500	S1—O14	1.505 (3)
C115—C116	1.399 (6)	S2—O24	1.450 (3)
C115—H115	0.9500	S2—O23	1.475 (3)
C117—H11A	0.9800	S2—O21	1.496 (3)
C117—H11B	0.9800	S2—O22	1.494 (3)
C117—H11C	0.9800	O13A—C12A	1.42 (3)
C118—H11D	0.9800	O13A—H13A	0.8400
C118—H11E	0.9800	C12A—H12A	0.9900
C118—H11F	0.9800	C12A—H12B	0.9900
C119—C12A	1.48 (3)	C39—C32A	1.621 (13)
C119—C12B	1.54 (3)	C39—H39A	0.9900
C119—H11G	0.9900	C39—H39B	0.9900
C119—H11H	0.9900	C32A—O32A	1.367 (15)
O201—C201	1.209 (6)	C32A—H32A	0.9900
O202—C201	1.333 (6)	C32A—H32B	0.9900
O202—H202	0.8400	O32A—H3AO	0.8400
O203—C220	1.403 (6)	O13B—C12B	1.40 (2)
O203—H23O	0.8400	O13B—H13B	0.8400
N201—N202	1.324 (5)	C12B—H12C	0.9900
N201—C207	1.390 (6)	C12B—H12D	0.9900
N201—H21N	0.90 (5)	O32B—C32B	1.31 (2)
N202—C208	1.301 (6)	O32B—H3BO	0.8400
N203—C209	1.324 (6)	C32B—H32C	1.00 (2)
N203—C216	1.403 (6)	C32B—H32D	0.99 (2)
N203—C219	1.457 (6)		
			
C1—O2—H2O	109.5	C203—C204—C205	118.7 (5)
C20—O3—H3O	109.5	C203—C204—H204	120.6
N2—N1—C7	121.7 (4)	C205—C204—H204	120.6
N2—N1—H1N	121 (5)	C206—C205—C204	120.1 (5)
C7—N1—H1N	116 (4)	C206—C205—H205	119.9
C8—N2—N1	115.9 (4)	C204—C205—H205	119.9
C9—N3—C16	111.1 (4)	C205—C206—C207	120.9 (5)
C9—N3—C19	126.6 (4)	C205—C206—H206	119.5
C16—N3—C19	122.3 (4)	C207—C206—H206	119.5
O1—C1—O2	123.3 (4)	N201—C207—C206	120.8 (4)
O1—C1—C2	122.9 (4)	N201—C207—C202	119.4 (4)
O2—C1—C2	113.7 (4)	C206—C207—C202	119.7 (5)
C3—C2—C7	117.8 (4)	N202—C208—C209	117.7 (4)
C3—C2—C1	120.4 (4)	N202—C208—H208	121.2
C7—C2—C1	121.7 (4)	C209—C208—H208	121.2
C2—C3—C4	123.8 (4)	N203—C209—C208	123.9 (4)
C2—C3—H3	118.1	N203—C209—C210	110.9 (4)
C4—C3—H3	118.1	C208—C209—C210	125.2 (4)
C3—C4—C5	116.9 (5)	C209—C210—C217	112.9 (4)
C3—C4—H4	121.6	C209—C210—C211	100.0 (4)
C5—C4—H4	121.6	C217—C210—C211	111.2 (4)
C6—C5—C4	121.5 (4)	C209—C210—C218	111.4 (4)
C6—C5—H5	119.2	C217—C210—C218	111.9 (4)
C4—C5—H5	119.2	C211—C210—C218	108.7 (4)
C5—C6—C7	119.9 (4)	C212—C211—C216	120.5 (5)
C5—C6—H6	120.1	C212—C211—C210	130.7 (5)
C7—C6—H6	120.1	C216—C211—C210	108.6 (4)
N1—C7—C6	119.6 (4)	C211—C212—C213	118.2 (5)
N1—C7—C2	120.4 (4)	C211—C212—H212	120.9
C6—C7—C2	120.0 (4)	C213—C212—H212	120.9
N2—C8—C9	118.1 (4)	C214—C213—C212	121.4 (5)
N2—C8—H8	120.9	C214—C213—H213	119.3
C9—C8—H8	120.9	C212—C213—H213	119.3
N3—C9—C8	122.0 (4)	C213—C214—C215	120.8 (5)
N3—C9—C10	110.7 (4)	C213—C214—H214	119.6
C8—C9—C10	127.3 (4)	C215—C214—H214	119.6
C9—C10—C11	100.6 (4)	C216—C215—C214	116.4 (5)
C9—C10—C17	112.1 (4)	C216—C215—H215	121.8
C11—C10—C17	110.9 (4)	C214—C215—H215	121.8
C9—C10—C18	110.4 (4)	C211—C216—C215	122.7 (5)
C11—C10—C18	110.4 (4)	C211—C216—N203	109.7 (4)
C17—C10—C18	111.9 (4)	C215—C216—N203	127.6 (5)
C16—C11—C12	120.0 (4)	C210—C217—H21A	109.5
C16—C11—C10	109.2 (4)	C210—C217—H21B	109.5
C12—C11—C10	130.8 (4)	H21A—C217—H21B	109.5
C13—C12—C11	117.6 (5)	C210—C217—H21C	109.5
C13—C12—H12	121.2	H21A—C217—H21C	109.5
C11—C12—H12	121.2	H21B—C217—H21C	109.5
C12—C13—C14	122.4 (5)	C210—C218—H21D	109.5
C12—C13—H13	118.8	C210—C218—H21E	109.5
C14—C13—H13	118.8	H21D—C218—H21E	109.5
C13—C14—C15	120.7 (5)	C210—C218—H21F	109.5
C13—C14—H14	119.7	H21D—C218—H21F	109.5
C15—C14—H14	119.7	H21E—C218—H21F	109.5
C16—C15—C14	115.5 (5)	N203—C219—C220	108.7 (4)
C16—C15—H15	122.3	N203—C219—H21G	109.9
C14—C15—H15	122.3	C220—C219—H21G	109.9
C15—C16—C11	123.8 (4)	N203—C219—H21H	109.9
C15—C16—N3	127.8 (4)	C220—C219—H21H	109.9
C11—C16—N3	108.4 (4)	H21G—C219—H21H	108.3
C10—C17—H17A	109.5	O203—C220—C219	111.8 (5)
C10—C17—H17B	109.5	O203—C220—H22A	109.3
H17A—C17—H17B	109.5	C219—C220—H22A	109.3
C10—C17—H17C	109.5	O203—C220—H22B	109.3
H17A—C17—H17C	109.5	C219—C220—H22B	109.3
H17B—C17—H17C	109.5	H22A—C220—H22B	107.9
C10—C18—H18A	109.5	C301—O302—H302	109.5
C10—C18—H18B	109.5	N302—N301—C307	119.8 (4)
H18A—C18—H18B	109.5	N302—N301—H31N	120 (3)
C10—C18—H18C	109.5	C307—N301—H31N	120 (3)
H18A—C18—H18C	109.5	C308—N302—N301	115.5 (4)
H18B—C18—H18C	109.5	C309—N303—C316	111.5 (4)
N3—C19—C20	112.9 (4)	C309—N303—C39	127.2 (4)
N3—C19—H19A	109.0	C316—N303—C39	120.9 (4)
C20—C19—H19A	109.0	O301—C301—O302	124.1 (4)
N3—C19—H19B	109.0	O301—C301—C302	123.3 (4)
C20—C19—H19B	109.0	O302—C301—C302	112.4 (4)
H19A—C19—H19B	107.8	C303—C302—C307	117.4 (4)
O3—C20—C19	113.8 (4)	C303—C302—C301	120.9 (4)
O3—C20—H20A	108.8	C307—C302—C301	121.7 (4)
C19—C20—H20A	108.8	C304—C303—C302	123.4 (4)
O3—C20—H20B	108.8	C304—C303—H303	118.3
C19—C20—H20B	108.8	C302—C303—H303	118.3
H20A—C20—H20B	107.7	C303—C304—C305	117.8 (5)
C101—O102—H102	109.5	C303—C304—H304	121.1
N102—N101—C107	120.0 (4)	C305—C304—H304	121.1
N102—N101—H11N	122 (3)	C306—C305—C304	121.1 (5)
C107—N101—H11N	118 (3)	C306—C305—H305	119.4
C108—N102—N101	117.1 (4)	C304—C305—H305	119.4
C109—N103—C116	110.1 (3)	C305—C306—C307	119.8 (5)
C109—N103—C119	126.4 (4)	C305—C306—H306	120.1
C116—N103—C119	123.0 (4)	C307—C306—H306	120.1
O101—C101—O102	123.2 (5)	C306—C307—C302	120.2 (4)
O101—C101—C102	122.4 (5)	C306—C307—N301	119.7 (4)
O102—C101—C102	114.5 (5)	C302—C307—N301	120.1 (4)
C103—C102—C107	117.7 (4)	N302—C308—C309	116.7 (4)
C103—C102—C101	119.7 (4)	N302—C308—H308	121.6
C107—C102—C101	122.6 (5)	C309—C308—H308	121.6
C104—C103—C102	122.1 (5)	N303—C309—C308	122.1 (4)
C104—C103—H103	119.0	N303—C309—C310	109.5 (4)
C102—C103—H103	119.0	C308—C309—C310	128.3 (4)
C103—C104—C105	119.4 (5)	C311—C310—C317	112.3 (4)
C103—C104—H104	120.3	C311—C310—C309	101.1 (4)
C105—C104—H104	120.3	C317—C310—C309	111.3 (4)
C106—C105—C104	120.7 (5)	C311—C310—C318	110.6 (4)
C106—C105—H105	119.6	C317—C310—C318	110.9 (4)
C104—C105—H105	119.6	C309—C310—C318	110.3 (4)
C105—C106—C107	121.1 (5)	C312—C311—C316	119.7 (4)
C105—C106—H106	119.5	C312—C311—C310	130.1 (4)
C107—C106—H106	119.5	C316—C311—C310	110.2 (4)
N101—C107—C106	122.0 (4)	C311—C312—C313	117.8 (5)
N101—C107—C102	119.0 (4)	C311—C312—H312	121.1
C106—C107—C102	119.0 (5)	C313—C312—H312	121.1
N102—C108—C109	117.7 (4)	C314—C313—C312	120.8 (5)
N102—C108—H108	121.1	C314—C313—H313	119.6
C109—C108—H108	121.1	C312—C313—H313	119.6
N103—C109—C108	122.4 (4)	C315—C314—C313	121.8 (5)
N103—C109—C110	110.5 (3)	C315—C314—H314	119.1
C108—C109—C110	127.1 (4)	C313—C314—H314	119.1
C111—C110—C109	100.9 (3)	C314—C315—C316	116.9 (5)
C111—C110—C118	110.9 (4)	C314—C315—H315	121.5
C109—C110—C118	110.5 (4)	C316—C315—H315	121.5
C111—C110—C117	112.0 (4)	C315—C316—C311	123.0 (4)
C109—C110—C117	111.4 (4)	C315—C316—N303	129.4 (4)
C118—C110—C117	110.8 (4)	C311—C316—N303	107.7 (4)
C112—C111—C116	119.1 (4)	C310—C317—H31A	109.5
C112—C111—C110	131.3 (4)	C310—C317—H31B	109.5
C116—C111—C110	109.7 (4)	H31A—C317—H31B	109.5
C111—C112—C113	119.3 (5)	C310—C317—H31C	109.5
C111—C112—H112	120.4	H31A—C317—H31C	109.5
C113—C112—H112	120.4	H31B—C317—H31C	109.5
C114—C113—C112	120.9 (5)	C310—C318—H31D	109.5
C114—C113—H113	119.6	C310—C318—H31E	109.5
C112—C113—H113	119.6	H31D—C318—H31E	109.5
C113—C114—C115	120.9 (5)	C310—C318—H31F	109.5
C113—C114—H114	119.5	H31D—C318—H31F	109.5
C115—C114—H114	119.5	H31E—C318—H31F	109.5
C114—C115—C116	117.2 (4)	O12—S1—O11	111.7 (2)
C114—C115—H115	121.4	O12—S1—O13	110.4 (2)
C116—C115—H115	121.4	O11—S1—O13	109.16 (19)
C111—C116—C115	122.7 (4)	O12—S1—O14	109.9 (2)
C111—C116—N103	108.7 (4)	O11—S1—O14	107.74 (19)
C115—C116—N103	128.6 (4)	O13—S1—O14	107.9 (2)
C110—C117—H11A	109.5	O24—S2—O23	111.2 (2)
C110—C117—H11B	109.5	O24—S2—O21	108.6 (2)
H11A—C117—H11B	109.5	O23—S2—O21	109.7 (2)
C110—C117—H11C	109.5	O24—S2—O22	110.1 (2)
H11A—C117—H11C	109.5	O23—S2—O22	108.5 (2)
H11B—C117—H11C	109.5	O21—S2—O22	108.7 (2)
C110—C118—H11D	109.5	C12A—O13A—H13A	109.5
C110—C118—H11E	109.5	O13A—C12A—C119	118.4 (19)
H11D—C118—H11E	109.5	O13A—C12A—H12A	107.7
C110—C118—H11F	109.5	C119—C12A—H12A	107.7
H11D—C118—H11F	109.5	O13A—C12A—H12B	107.7
H11E—C118—H11F	109.5	C119—C12A—H12B	107.7
N103—C119—C12A	112.2 (11)	H12A—C12A—H12B	107.1
N103—C119—C12B	108.4 (10)	N303—C39—C32A	105.8 (6)
N103—C119—H11G	109.2	N303—C39—H39A	110.6
C12A—C119—H11G	109.2	C32A—C39—H39A	110.6
N103—C119—H11H	109.2	N303—C39—H39B	110.6
C12A—C119—H11H	109.2	C32A—C39—H39B	110.6
H11G—C119—H11H	107.9	H39A—C39—H39B	108.7
C201—O202—H202	109.5	O32A—C32A—C39	115.6 (8)
C220—O203—H23O	109.5	O32A—C32A—H32A	108.4
N202—N201—C207	119.7 (4)	C39—C32A—H32A	108.4
N202—N201—H21N	125 (3)	O32A—C32A—H32B	108.4
C207—N201—H21N	115 (3)	C39—C32A—H32B	108.4
C208—N202—N201	117.7 (4)	H32A—C32A—H32B	107.4
C209—N203—C216	110.6 (4)	C32A—O32A—H3AO	109.5
C209—N203—C219	127.8 (4)	C12B—O13B—H13B	109.5
C216—N203—C219	121.0 (4)	O13B—C12B—C119	112.3 (17)
O201—C201—O202	124.9 (4)	O13B—C12B—H12C	109.2
O201—C201—C202	123.7 (4)	C119—C12B—H12C	109.2
O202—C201—C202	111.4 (4)	O13B—C12B—H12D	109.2
C203—C202—C207	117.6 (4)	C119—C12B—H12D	109.2
C203—C202—C201	121.4 (4)	H12C—C12B—H12D	107.9
C207—C202—C201	121.0 (4)	C32B—O32B—H3BO	109.5
C204—C203—C202	122.9 (5)	O32B—C32B—H32C	108.3 (19)
C204—C203—H203	118.5	O32B—C32B—H32D	109.2 (17)
C202—C203—H203	118.5	H32C—C32B—H32D	107 (2)
			
C7—N1—N2—C8	174.7 (4)	O201—C201—C202—C203	173.4 (5)
O1—C1—C2—C3	−175.7 (5)	O202—C201—C202—C203	−8.3 (6)
O2—C1—C2—C3	8.1 (6)	O201—C201—C202—C207	−7.0 (7)
O1—C1—C2—C7	2.9 (7)	O202—C201—C202—C207	171.2 (4)
O2—C1—C2—C7	−173.3 (4)	C207—C202—C203—C204	−0.7 (7)
C7—C2—C3—C4	2.2 (7)	C201—C202—C203—C204	178.9 (5)
C1—C2—C3—C4	−179.1 (4)	C202—C203—C204—C205	−0.6 (8)
C2—C3—C4—C5	−1.8 (8)	C203—C204—C205—C206	1.4 (8)
C3—C4—C5—C6	1.5 (8)	C204—C205—C206—C207	−0.8 (8)
C4—C5—C6—C7	−1.9 (8)	N202—N201—C207—C206	−6.1 (7)
N2—N1—C7—C6	7.5 (7)	N202—N201—C207—C202	170.8 (4)
N2—N1—C7—C2	−172.3 (4)	C205—C206—C207—N201	176.4 (5)
C5—C6—C7—N1	−177.5 (5)	C205—C206—C207—C202	−0.6 (8)
C5—C6—C7—C2	2.3 (7)	C203—C202—C207—N201	−175.7 (4)
C3—C2—C7—N1	177.4 (4)	C201—C202—C207—N201	4.7 (7)
C1—C2—C7—N1	−1.2 (7)	C203—C202—C207—C206	1.3 (7)
C3—C2—C7—C6	−2.4 (7)	C201—C202—C207—C206	−178.3 (4)
C1—C2—C7—C6	178.9 (4)	N201—N202—C208—C209	176.8 (4)
N1—N2—C8—C9	−178.9 (4)	C216—N203—C209—C208	176.3 (4)
C16—N3—C9—C8	−178.6 (4)	C219—N203—C209—C208	5.3 (7)
C19—N3—C9—C8	−0.8 (7)	C216—N203—C209—C210	−2.8 (5)
C16—N3—C9—C10	1.9 (5)	C219—N203—C209—C210	−173.8 (4)
C19—N3—C9—C10	179.7 (4)	N202—C208—C209—N203	−173.6 (4)
N2—C8—C9—N3	178.1 (4)	N202—C208—C209—C210	5.4 (7)
N2—C8—C9—C10	−2.4 (7)	N203—C209—C210—C217	−117.6 (4)
N3—C9—C10—C11	−0.9 (5)	C208—C209—C210—C217	63.3 (6)
C8—C9—C10—C11	179.6 (4)	N203—C209—C210—C211	0.7 (5)
N3—C9—C10—C17	−118.7 (4)	C208—C209—C210—C211	−178.4 (4)
C8—C9—C10—C17	61.8 (6)	N203—C209—C210—C218	115.5 (4)
N3—C9—C10—C18	115.8 (4)	C208—C209—C210—C218	−63.6 (6)
C8—C9—C10—C18	−63.7 (6)	C209—C210—C211—C212	176.3 (5)
C9—C10—C11—C16	−0.5 (5)	C217—C210—C211—C212	−64.2 (7)
C17—C10—C11—C16	118.3 (4)	C218—C210—C211—C212	59.5 (7)
C18—C10—C11—C16	−117.2 (4)	C209—C210—C211—C216	1.7 (5)
C9—C10—C11—C12	−178.1 (5)	C217—C210—C211—C216	121.2 (4)
C17—C10—C11—C12	−59.4 (7)	C218—C210—C211—C216	−115.2 (5)
C18—C10—C11—C12	65.2 (7)	C216—C211—C212—C213	−1.5 (8)
C16—C11—C12—C13	3.1 (7)	C210—C211—C212—C213	−175.6 (5)
C10—C11—C12—C13	−179.4 (5)	C211—C212—C213—C214	1.4 (8)
C11—C12—C13—C14	−3.3 (8)	C212—C213—C214—C215	−1.4 (8)
C12—C13—C14—C15	2.2 (8)	C213—C214—C215—C216	1.4 (8)
C13—C14—C15—C16	−0.8 (7)	C212—C211—C216—C215	1.7 (7)
C14—C15—C16—C11	0.7 (7)	C210—C211—C216—C215	176.9 (4)
C14—C15—C16—N3	179.0 (4)	C212—C211—C216—N203	−178.7 (4)
C12—C11—C16—C15	−1.9 (7)	C210—C211—C216—N203	−3.4 (5)
C10—C11—C16—C15	−179.8 (4)	C214—C215—C216—C211	−1.6 (7)
C12—C11—C16—N3	179.5 (4)	C214—C215—C216—N203	178.8 (4)
C10—C11—C16—N3	1.5 (5)	C209—N203—C216—C211	4.0 (5)
C9—N3—C16—C15	179.3 (5)	C219—N203—C216—C211	175.7 (4)
C19—N3—C16—C15	1.3 (7)	C209—N203—C216—C215	−176.4 (5)
C9—N3—C16—C11	−2.2 (5)	C219—N203—C216—C215	−4.7 (7)
C19—N3—C16—C11	179.9 (4)	C209—N203—C219—C220	90.9 (6)
C9—N3—C19—C20	−84.7 (6)	C216—N203—C219—C220	−79.3 (5)
C16—N3—C19—C20	92.9 (5)	N203—C219—C220—O203	−64.6 (6)
N3—C19—C20—O3	−65.3 (5)	C307—N301—N302—C308	178.5 (4)
C107—N101—N102—C108	177.1 (4)	O301—C301—C302—C303	179.3 (5)
O101—C101—C102—C103	−179.0 (5)	O302—C301—C302—C303	4.8 (6)
O102—C101—C102—C103	2.0 (7)	O301—C301—C302—C307	0.4 (7)
O101—C101—C102—C107	1.1 (8)	O302—C301—C302—C307	−174.0 (4)
O102—C101—C102—C107	−178.0 (4)	C307—C302—C303—C304	3.4 (7)
C107—C102—C103—C104	0.4 (7)	C301—C302—C303—C304	−175.5 (5)
C101—C102—C103—C104	−179.5 (4)	C302—C303—C304—C305	1.3 (8)
C102—C103—C104—C105	2.4 (7)	C303—C304—C305—C306	−3.1 (8)
C103—C104—C105—C106	−2.7 (8)	C304—C305—C306—C307	0.2 (8)
C104—C105—C106—C107	0.3 (8)	C305—C306—C307—C302	4.6 (7)
N102—N101—C107—C106	9.5 (7)	C305—C306—C307—N301	−176.7 (5)
N102—N101—C107—C102	−170.5 (4)	C303—C302—C307—C306	−6.3 (7)
C105—C106—C107—N101	−177.5 (5)	C301—C302—C307—C306	172.6 (4)
C105—C106—C107—C102	2.5 (7)	C303—C302—C307—N301	175.0 (4)
C103—C102—C107—N101	177.2 (4)	C301—C302—C307—N301	−6.1 (7)
C101—C102—C107—N101	−2.9 (7)	N302—N301—C307—C306	10.7 (6)
C103—C102—C107—C106	−2.8 (7)	N302—N301—C307—C302	−170.6 (4)
C101—C102—C107—C106	177.2 (4)	N301—N302—C308—C309	−180.0 (4)
N101—N102—C108—C109	−178.7 (4)	C316—N303—C309—C308	−178.1 (4)
C116—N103—C109—C108	179.7 (4)	C39—N303—C309—C308	−6.1 (7)
C119—N103—C109—C108	−8.2 (7)	C316—N303—C309—C310	2.0 (5)
C116—N103—C109—C110	0.4 (5)	C39—N303—C309—C310	174.0 (4)
C119—N103—C109—C110	172.6 (4)	N302—C308—C309—N303	176.3 (4)
N102—C108—C109—N103	177.8 (4)	N302—C308—C309—C310	−3.8 (7)
N102—C108—C109—C110	−3.1 (7)	N303—C309—C310—C311	−1.4 (5)
N103—C109—C110—C111	−0.2 (5)	C308—C309—C310—C311	178.8 (4)
C108—C109—C110—C111	−179.4 (4)	N303—C309—C310—C317	118.1 (4)
N103—C109—C110—C118	−117.7 (4)	C308—C309—C310—C317	−61.8 (6)
C108—C109—C110—C118	63.1 (6)	N303—C309—C310—C318	−118.4 (4)
N103—C109—C110—C117	118.8 (4)	C308—C309—C310—C318	61.7 (6)
C108—C109—C110—C117	−60.4 (6)	C317—C310—C311—C312	62.6 (7)
C109—C110—C111—C112	−177.8 (5)	C309—C310—C311—C312	−178.7 (5)
C118—C110—C111—C112	−60.7 (7)	C318—C310—C311—C312	−61.9 (6)
C117—C110—C111—C112	63.6 (6)	C317—C310—C311—C316	−118.4 (4)
C109—C110—C111—C116	0.0 (5)	C309—C310—C311—C316	0.3 (5)
C118—C110—C111—C116	117.1 (4)	C318—C310—C311—C316	117.1 (4)
C117—C110—C111—C116	−118.6 (4)	C316—C311—C312—C313	0.0 (8)
C116—C111—C112—C113	1.5 (7)	C310—C311—C312—C313	179.0 (5)
C110—C111—C112—C113	179.1 (5)	C311—C312—C313—C314	−0.3 (8)
C111—C112—C113—C114	−0.8 (8)	C312—C313—C314—C315	−0.4 (8)
C112—C113—C114—C115	0.0 (8)	C313—C314—C315—C316	1.3 (8)
C113—C114—C115—C116	0.1 (7)	C314—C315—C316—C311	−1.6 (7)
C112—C111—C116—C115	−1.5 (7)	C314—C315—C316—N303	179.6 (5)
C110—C111—C116—C115	−179.6 (4)	C312—C311—C316—C315	0.9 (8)
C112—C111—C116—N103	178.4 (4)	C310—C311—C316—C315	−178.2 (4)
C110—C111—C116—N103	0.3 (5)	C312—C311—C316—N303	180.0 (4)
C114—C115—C116—C111	0.7 (7)	C310—C311—C316—N303	0.8 (5)
C114—C115—C116—N103	−179.1 (4)	C309—N303—C316—C315	177.1 (5)
C109—N103—C116—C111	−0.4 (5)	C39—N303—C316—C315	4.5 (7)
C119—N103—C116—C111	−172.9 (4)	C309—N303—C316—C311	−1.8 (5)
C109—N103—C116—C115	179.4 (5)	C39—N303—C316—C311	−174.4 (4)
C119—N103—C116—C115	7.0 (7)	N103—C119—C12A—O13A	−64.2 (19)
C109—N103—C119—C12A	−88.8 (12)	C12B—C119—C12A—O13A	−122 (18)
C116—N103—C119—C12A	82.4 (12)	C309—N303—C39—C32A	−88.8 (8)
C109—N103—C119—C12B	−82.7 (11)	C316—N303—C39—C32A	82.6 (7)
C116—N103—C119—C12B	88.5 (11)	N303—C39—C32A—O32A	154.6 (10)
C207—N201—N202—C208	−178.7 (4)	N103—C119—C12B—O13B	61.0 (18)

### Hydrogen-bond geometry (Å, º)

**Table:**

*D*—H···*A*	*D*—H	H···*A*	*D*···*A*	*D*—H···*A*
O32*A*—H3A0···O101	0.84	1.94	2.644 (8)	140
O2—H2*O*···O14	0.84	1.66	2.457 (5)	158
N1—H1*N*···O1	0.70 (5)	2.08 (5)	2.652 (5)	139 (6)
O3—H3*O*···O13^i^	0.84	1.97	2.792 (5)	165
N101—H11*N*···O101	0.97 (5)	1.90 (5)	2.642 (6)	132 (4)
N201—H21*N*···O201	0.90 (5)	1.89 (6)	2.626 (6)	138 (5)
O203—H23*O*···O701^ii^	0.84	1.95	2.741 (6)	157
N301—H31*N*···O301	0.96 (5)	1.95 (5)	2.654 (5)	129 (4)
O102—H102···O21^iii^	0.84	1.68	2.509 (5)	169
O202—H202···O13^iv^	0.84	1.81	2.565 (5)	149
O302—H302···O22^v^	0.84	1.77	2.562 (6)	157
C8—H8···O11^vi^	0.95	2.55	3.402 (6)	150
C12*B*—H12*C*···O301	0.99	2.41	3.37 (2)	164
C15—H15···O12^i^	0.95	2.29	3.143 (6)	149
C19—H19*B*···O11^vi^	0.99	2.39	3.357 (7)	166
C20—H20*B*···O201^vi^	0.99	2.41	3.343 (6)	157
C218—H21*E*···O13*B*	0.98	2.44	3.193 (8)	133
C219—H21*H*···O1	0.99	2.41	3.072 (6)	124
C220—H22*B*···O1^vi^	0.99	2.48	3.456 (6)	168
C39—H39*A*···O101^iii^	0.99	2.55	3.248 (6)	128
C39—H39*B*···O23	0.99	2.53	3.514 (7)	175
C108—H108···O24	0.95	2.26	3.073 (6)	143
C114—H114···O102^vii^	0.95	2.54	3.323 (7)	139
C115—H115···O23^v^	0.95	2.39	3.202 (7)	143
C205—H205···O21^v^	0.95	2.57	3.359 (7)	141
C208—H208···O12	0.95	2.26	3.119 (5)	150
C212—H212···O80*A*	0.95	2.60	3.486 (10)	156
C215—H215···O11^vi^	0.95	2.27	3.137 (7)	152
C306—H306···O203^viii^	0.95	2.36	3.154 (7)	140
C308—H308···O23	0.95	2.39	3.278 (6)	155
C315—H315···O24^iii^	0.95	2.26	3.150 (6)	156

Symmetry codes: (i) *x*, *y*+1, *z*; (ii) *x*−1, *y*, *z*; (iii) −*x*+1, −*y*+1, −*z*+1; (iv) −*x*, −*y*, −*z*; (v) −*x*+1, −*y*, −*z*+1; (vi) −*x*, −*y*+1, −*z*; (vii) *x*, *y*−1, *z*; (viii) *x*+1, *y*, *z*.
